# Evidence that Gag facilitates HIV-1 envelope association both in GPI-enriched plasma membrane and detergent resistant membranes and facilitates envelope incorporation onto virions in primary CD4^+ ^T cells

**DOI:** 10.1186/1743-422X-7-3

**Published:** 2010-01-08

**Authors:** Ajit Patil, Archana Gautam, Jayanta Bhattacharya

**Affiliations:** 1 Division of Molecular Virology, National AIDS Research Institute, G-73 MIDC, Bhosari, Pune-411026, India

## Abstract

HIV-1 particle assembly mediated by viral Gag protein occurs predominantly at plasma membrane. While colocalization of HIV-1 envelope with lipid rich microenvironment have been shown in T cells, the significance of viral proteins modulating envelope association in such microdomains in plasma membrane enriched in glycosylphosphatidylinositol-anchored proteins in primary CD4^+ ^T cells that are natural targets of HIV-1 is poorly understood. Here we show that in primary CD4^+ ^T cells that are natural targets of HIV-1 *in vivo*, Gag modulates HIV-1 envelope association with GM1 ganglioside and CD59 rich cellular compartments as well as with detergent resistant membranes. Our data strengthen evidence that Gag-Env interaction is important in envelope association with lipid rafts containing GPI-anchored proteins for efficient assembly onto mature virions resulting in productive infection of primary CD4^+ ^T cells.

## Findings

Human Immunodeficiency Virus Type 1 (HIV-1) has been shown to assemble via specialized plasma membrane domains popularly known as lipid rafts [1-6], which are rich in cholesterol and sphingomyelin within an ordered structure and plays important role in cell signaling [7]. Rafts are believed to play an important role towards facilitating HIV-1 assembly particularly exploiting acylated residues in viral Gag [1,3,5] and envelope (Env) [8,9]. However, the precise mechanism by which lipid rafts functions in targeting viral Env and Gag to the plasma membrane in infected T cells and facilitate assembly is not clearly understood. Previously, Jolly and Sattentau [10] have shown that raft integrity is critical for Env and Gag co-clustering and assembly in T-cell conjugates. Thus, mere presence of non raft proteins such as CD45 phosphatase in HIV-1 envelope glycoprotein while abundant incorporation of raft lipid components such as ganglioside GM1, glycosylphosphatidylinositol (GPI)-anchored proteins Thy-1 and CD59 strongly suggest that HIV-1 specifically buds from rafts [5,11]. A stable interaction between intracellular Pr55Gag and the gp41 cytoplasmic domain of envelope [12] was shown to be important for envelope association with detergent resistant membranes, incorporation onto virions and infectivity [13,14]. The precise sequence by which envelope utilizes cellular machinery in migrating towards the site of viral assembly is not clearly understood. Glycoproteins of several enveloped viruses, have been found to contain lipid moieties [15,16] and has generated notion on the importance of lipid rafts as a docking site for the assembly of enveloped viruses [17-22]. Association of HIV-1 envelope with polarized lipid raft markers GM1 and CD59 was shown to influence transmission between T cells [10]. Gag has been shown to play an important role in envelope assembly onto virions, notably by interaction of its p17 matrix domain with gp41 cytoplasmic domain of envelope [14,23-25]. While HIV Gag intrinsically associates with detergent resistant membranes (DRMs) [5,26], influenza virus M1 protein transport to DRMs depends on co-expression of HA and NA glycoproteins [27]. Likewise, the association of Sendai virus M protein in DRM is dependent on expression of F or HN protein [28], while the Rous sarcoma virus M protein requires expression with the F protein for DRM association [29]. In contrast, the Measles virus M protein has been shown to associate DRMs intrinsically independent of other viral proteins [30]. Motifs in gp41 cytoplasmic domain regulating association of HIV-1 envelope protein with DRM [8] and this phenomenon is Gag dependent in a T cell line [13] was previously reported. However, it was not known whether this phenomenon is cell type-dependent and if it differs between cell lines and those that are natural targets *in vivo*. Moreover, whether DRM association of envelope corroborates with their ability to traffic to classical lipid rafts was also not known. In the present study we investigated the role of Gag in intracellular transport of HIV-1 envelope into well defined GPI-anchored proteins such as CD59 and GM1 (monosialotetrahexosylganglioside) and its relevance of envelope assembly onto budding virions in primary CD4^+ ^T cells. Precisely, we examined whether a point mutation (L30E) in matrix domain of Gag known for disrupting Env incorporation [23,31] affects envelope trafficking to CD59+ compartment in primary CD4^+ ^T cells and if this phenomenon has any association with cell-free infectivity in primary CD4^+ ^T cells.

**Figure 1:**
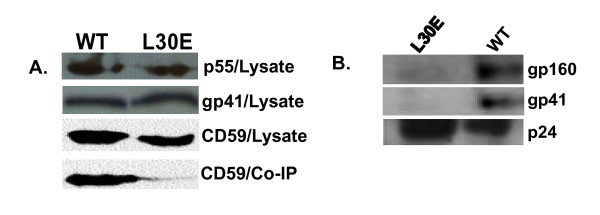
**A. L30E Gag confers defective association of Env with CD59-enriched membranes in primary CD4^+ ^T cells**. Primary CD4^+ ^T cells infected with VSV-G pseudotyped pNL4.3 and pNL4.3 (L30E) were lyzed and intracellular p55, gp41 and CD59 contents were measured in total cell lysates (as shown here in upper panels). Cell lysates expressing comparable p55 and gp41 were immunoprecipitated with gp41 antibodies 2F5 and 4E10 as described in the Materials and Methods resolved in 12.5% SDS-PAGE and electrophoretically transferred onto PVDF membrane. The level of CD59 co-immunoprecipitated with envelope was detected by Western blotting using monoclonal anti-CD59 antibody. **B**. Defective incorporation of HIV-1 envelope proteins onto virus particles due to L30E Gag substitution. Equal amounts of cell free virus particles were resolved in SDS-PAGE and Western blotting done by monoclonal antibodies to gp41 and p24 as described in the text. Note that with L30E gag mutation, envelope incorporation onto virions is severely abrogated.

We first examined whether a point mutation (L30E) in matrix domain of Gag previously reported to abrogate envelope incorporation, infectivity [23] and DRM association [13] in cell lines affect infectivity and modulate envelope association with CD59-enriched compartment in primary CD4^+ ^T cells which are predominantly the natural targets *in vivo *during entire course of HIV-1 infection. CD59 marker was selected as it is linked with phosphatidylinositol and segregates into rafts. Primary CD4^+ ^T cells were purified by negative selection from whole blood using RosetteSep^® ^Kit (Stem Cell Technologies Inc.) following manufacturer's protocol. Briefly, CD4^+ ^T Cell Enrichment Cocktail was added at a final concentration of 50 μl/ml of whole blood and incubated at room temperature for 20 min. The mixture was diluted with RPMI/2% Fetal Bovine Serum (GIBCO, Inc), layered onto Ficoll Hypaque (Sigma, Inc) and centrifuged for 20 min at 1200 × g at room temperature. The enriched CD4^+ ^T cells in the interface of density medium and plasma yielded approximately 98% purity; subsequently stimulated with phytohemagglutinin (0.5 ug/ml) and interleukin-2 (10-20 U/ml) before infection with virus stocks. Primary CD4^+ ^T cells were infected with equal infectivity titers of VSV-G pseudotyped pNL4.3 WT and pNL4.3 L30E viruses and the cell lysates made from CD4^+ ^T cells infected with wild type and L30E expressing equal amounts of p24 and gp41 were incubated with anti-gp41 monoclonal antibodies to immuno-precipitate Env. Briefly, CD4^+ ^T cells were lyzed with 1% Triton X-100 in PBS (pH 7.4), and incubated with 1:1 mixture of gp41 monoclonal antibodies 2F5 and 4E10 (1:1000 dilution) for 4 hours at 4°C followed by additional 1 hour incubation with Protein A/G beads (Pierce Inc). Lysates were further washed with cold PBS and were resolved in 12% SDS-PAGE under denaturing conditions. Equal amounts of immunoprecipitated materials were subjected to 12% SDS-PAGE under denaturing conditions [32], transferred onto PVDF membranes and subsequently Western blot assay done with anti-human CD59 antibody (at 1: 1000 dilution) to assess the ability of Env association with CD59-enriched compartment. As shown in Figure 1A, due to L30E mutation in Gag, Env failed to recruit CD59 in contrast to pNL4.3 wild type. Our data indicate that the mutation in Gag MA region (L30E) restricts the interaction between Gag and Env resulting in down modulation of envelope trafficking to GPI-anchored membranes in primary CD4^+ ^T cells such as CD59. Moreover, in order to assess the effect of L30E mutation in p17 gag on HIV-1 envelope incorporation on virions in primary CD4^+ ^T cells, cell-free virus pellets were obtained by centrifugation as described previously [13]. Equal amounts of virus particles (p24) were resolved in SDS-PAGE under denaturing conditions followed by Western blotting using monoclonal antibodies to p24 (183-H12-5C) and gp41 (1:1 of 2F5 and 4E10). As shown in Figure 1B, L30E substitution in p17 Gag was found to drastically affect envelope incorporation onto virus particles in CD4^+ ^T cells as expected.

**Figure 2:**
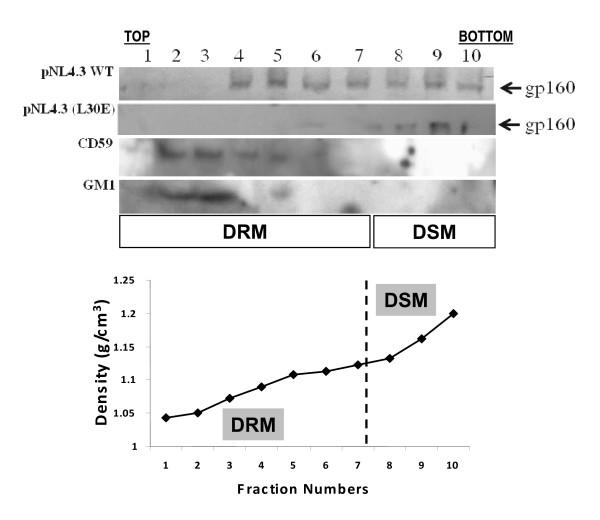
**Envelope association with DRM in primary CD4^+ ^T cells**. A. CD4^+ ^T cells infected with VSV-G pseudotyped pNL4.3 and pNL4.3 (L30E) were treated with cold Triton-X 100 and fractionated in sucrose density gradients as described in the text. Gradient fractions were subsequently probed for envelope with gp41 antibodies 2F5 and 4E10, anti-human CD59 for CD59 and CTxB-HRP for GM1 by SDS-PAGE followed by Western blotting. B. Density (g/cm^3^) of fractions showing DRM and DSM (detergent soluble membrane) fractions [39].

We next assessed if there is any correlation between disruptions of envelope association with CD59+ compartment in primary CD4^+ ^T cells and its association with DRM of the same cell type. The DRM assay was carried out essentially as described previously [8]. Briefly, primary CD4^+ ^T cells infected with VSV-G pseudotyped pNL4.3 WT and pNL4.3 (L30E) were lyzed with cold 0.5% Triton-X-100 and fractionated through sucrose density gradient centrifugation in an ultracentrifuge (Beckman Coulter Inc) at 1,00,000 × g for 8-12 hours at 4°C. Ten equal fractions were collected and were examined for presence of gp160, CD59 and GM-1 ganglioside by Western blot using human anti-gp41 monoclonal antibody (2F5) [31] and 4E10 [33], mouse anti-human CD59 (BD Biosciences Inc) and cholera toxin conjugated to horse radish peroxidase (HRP) (Sigma, Inc.) respectively. As shown in Figure 2, L30E mutation in Gag restricted envelope association with DRM fractions of CD4^+ ^T cells in contrast to the wild type. Our results were further substantiated by the presence of both CD59 and GM1 in DRM fractions under same experimental conditions.

In summary, we show the modulatory role of Gag on envelope association with lipid enriched micro domain that play an important role in transmission by modulating envelope assembly onto virus particles in primary CD4^+ ^T cells. Despite its close proximity, DRMs isolated from cells may not necessarily represent the preexisting rafts in living cells. [34,35]. Hence, GPI anchored proteins because of their raftofilcity often are regarded as best choice for studying raft association and targeting [36]. In our present study, in addition to investigate role of gag in envelope association with DRM, we attempted to look into GPI-anchored proteins for their high affinity towards lipid raft association [35,37]. The lipid-binding B subunit of cholera toxin (CTxB), recognizing GM1 at cell surface in plasma membrane, was used to study the envelope transport into lipid rafts. Like other GPI-anchored proteins, these markers were also known to be associated with DRM [38]. Our data showed that abrogation of Gag-Env interaction down modulated envelope transport into CD59 positive compartment in primary CD4^+ ^T cells and also failed to associate with DRM fractions. While viral precursors Gag and Gag-Pol are synthesized by polysomes in cellular cytoplasm, the oligomeric envelope protein is synthesized in endoplasmic reticulum and post-translationally modulated in the Golgi apparatus, traverses into the secretory pathway towards assembling onto budding virions. We envisage that Gag by acting as cargo transport intermediate carry envelope protein through trans-golgi route sorting into domains of lipid rich plasma membrane enable them assemble onto budding virions in primary CD4^+ ^T cells.

## List of abbreviations

DRM: detergent resistant membrane; GM1: monosialotetrahexosylganglioside; GPI: glycosylphosphatidylinositol; GHOST: human osteosarcoma cells expressing CD4 and green fluorescent protein; CTxB: cholera toxin-b subunit.

## Competing interests

The authors declare that they have no competing interests.

## Authors' contributions

JB has designed the study; AP and AG have performed the experiments; AP and AG helped JB in writing the manuscript. All the authors have read and approved the final manuscript.
